# *Killer-cell Immunoglobulin-like Receptor* (KIR) gene profiles modify HIV disease course, not HIV acquisition in South African women

**DOI:** 10.1186/s12879-016-1361-1

**Published:** 2016-01-25

**Authors:** V. Naranbhai, D. de Assis Rosa, L. Werner, R. Moodley, H. Hong, A. Kharsany, K. Mlisana, S. Sibeko, N. Garrett, D. Chopera, W. H. Carr, Q. Abdool Karim, A. V. S. Hill, S. S. Abdool Karim, M. Altfeld, C. M. Gray, T. Ndung’u

**Affiliations:** 1 Centre for the AIDS Programme of Research in South Africa (CAPRISA), University of KwaZulu-Natal, Durban, South Africa; 2 Wellcome Trust Centre for Human Genetics, Nuffield Department of Medicine, University of Oxford, Oxford, UK; 3 HIV Pathogenesis Programme, University of KwaZulu-Natal, Durban, South Africa; 4 National Institute of Communicable Diseases, Sandringham, South Africa; 5 University of the Witwatersrand, Johannesburg, South Africa; 6 University of Cape Town, Cape Town, South Africa; 7 City University of New York - Medgar Evers College, New York, USA; 8 Ragon Institute of MGH, MIT and Harvard University, Boston, USA; 9 Mailman School of Public Health, Columbia University, New York, USA; 10 Leibniz Institute for Experimental Virology, Heinrich Pette Institute, Hamburg, Germany; 11 KwaZulu-Natal Research Institute for Tuberculosis and HIV, University of KwaZulu-Natal, Durban, South Africa; 12 Max Planck Institute for Infection Biology, Chariteplatz, D-10117 Berlin, Germany; 13 Division of Virology, School of Pathology, Faculty of Health Sciences, University of the Witwatersrand, Johannesburg, South Africa

**Keywords:** KIR, HLA, HIV, Acquisition, Viral control, Disease progression

## Abstract

**Background:**

Killer-cell Immunoglobulin-like Receptors(KIR) interact with Human Leukocyte Antigen(HLA) to modify natural killer- and T-cell function. KIR are implicated in HIV acquisition by small studies that have not been widely replicated. A role for KIR in HIV disease progression is more widely replicated and supported by functional studies.

**Methods:**

To assess the role of KIR and KIR ligands in HIV acquisition and disease course, we studied at-risk women in South Africa between 2004–2010. Logistic regression was used for nested case–control analysis of 154 women who acquired vs. 155 who did not acquire HIV, despite high exposure. Linear mixed-effects models were used for cohort analysis of 139 women followed prospectively for a median of 54 months (IQR 31–69) until 2014.

**Results:**

Neither KIR repertoires nor HLA alleles were associated with HIV acquisition. However, KIR haplotype BB was associated with lower viral loads (−0.44log10 copies/ml;SE = 0.18;p = 0.03) and higher CD4+ T-cell counts(+80 cells/μl;SE = 42;p = 0.04). This was largely explained by the protective effect of *KIR2DL2/KIR2DS2* on the B haplotype and reciprocal detrimental effect of *KIR2DL3* on the A haplotype.

**Conclusions:**

Although neither KIR nor HLA appear to have a role in HIV acquisition, our data are consistent with involvement of KIR2DL2 in HIV control. Additional studies to replicate these findings are indicated.

**Electronic supplementary material:**

The online version of this article (doi:10.1186/s12879-016-1361-1) contains supplementary material, which is available to authorized users.

## Background

An array of host genetic factors have been reported to alter HIV acquisition or markers of HIV disease progression [1, 2]. Identifying and understanding these genetic correlates may accelerate preventive and therapeutic efforts.

The most widely replicated correlates of HIV acquisition include homozygosity in the 32-base pair deletion in *CCR5* that reduces acquisition risk. Although, in comparison, greater effort has been focused on identifying correlates of HIV disease progression there are similarly only a handful of widely replicated findings. Natural Killer (NK) cell and CD8+ T-cell responses stand out as being reproducibly implicated in HIV control [3]. Concordantly, variation in *the Human Leukocyte Antigen* (HLA) and *Killer-cell Immunoglobulin-like Receptor* (KIR) loci, the two most polymorphic regions of the human genome that encode receptors involved in NK and CD8+ T-cell function, is associated with rates of HIV disease progression across several studies [2]. Genome-wide association studies identify HLA-B*57 alleles, and variants that affect HLA-C expression [4–6] as important modifiers of HIV viraemia, the former of which had been identified in many earlier candidate gene studies.

Natural Killer cells are amongst the earliest responders to viral infection and mediate protective responses by secreting pro-inflammatory cytokines and by direct cytolysis of infected cells. Their function is governed, at least in part, by the combinatorial array of inhibitory and activating receptors including the KIR, Leukocyte immunoglobulin-like receptors (LILR), the C-type lectin receptors-NKG2A-F, and the natural cytotoxic receptors (NCRs) -NKp30, NKp44 and NKp46. The dominant regulators of NK cell recognition of virus-infected cells are thought to be KIR, because these are the natural receptors for HLA class I [7]. Alteration in HLA expression and presentation of pathogen-derived or self-peptides is a common feature of many viral infections [8]. Diversity in *KIR* gene content, polymorphism and structural variation within the 14 *KIR* genes, and variation in expression confer additional variation in the ability of NK cells to identify and respond to virus-infected cells. KIRs show partial specificity in their recognition of HLA ligands: KIR3DL1 and KIR3DS1 recognise HLA-A and HLA-B molecules with the Bw4 epitope, KIR2DL1 recognises HLA-C molecules of the C2 group exclusively, KIR2DL3 recognises HLA-C of the C1 group exclusively and KIR2DL2 recognizes HLA-C of both C1 and C2 groups. The KIR genes segregate in two groups of haplotypes. Group A haplotypes consist of nine genes that encode predominantly inhibitory receptors, whereas group B haplotypes represent a more diverse collection of haplotypes based on gene content and contain more activating *KIRs* compared with haplotype A.

Several combinations of *KIR* and *HLA* have been associated with protection from HIV acquisition [9–13], though these findings are based on small sample sizes and none have been replicated in the literature. In contrast, a large study reported in 2002, found that *KIR3DS1* together with *HLA Bw4* alleles encoding Isoleucine at position 80 (80I) are associated with slower disease progression [5]. Several additional lines of evidence from subsequent observational and functional studies support a role for KIR in HIV control. NK cells are expanded in primary HIV infection [14], the expansion is modified by specific KIR and ligand repertoires [15], the degree of HLA C ligand expression associates with protection [4, 16] and viruses sequenced from individuals with specific *KIR* show evidence of ‘escape’ mutation at sites that appear to alter recognition by KIR [17]. As in infection with EBV [18], CMV [19], HCV or HTLV-1 [20], KIR have also been shown to modulate T-cell responses to HIV [21].

We studied the role of *HLA* and *KIR* on risk of HIV acquisition and course of HIV viraemia and CD4+ T-cell counts through the first 5–10 years of infection in South African women infected with HIV-1 clade C in a nested case–control and prospective cohort study respectively. In prior studies in this cohort we have reported that a) innate immune cell activation is associated with enhanced HIV acquisition [22]; b) HIV-directed NK cells secreting IFN-y are associated with reduced HIV acquisition risk [23]; c) HIV acquisition results in profound alteration in NK cell function [24] and d) the rate of HIV disease progression is more rapid than similar cohorts elsewhere [25, 26]. This current study strongly implicates *KIR2DL2*, belonging to the group B haplotype with HIV control.

## Methods

### Study design and cohort accrual

**Fig. 1:**
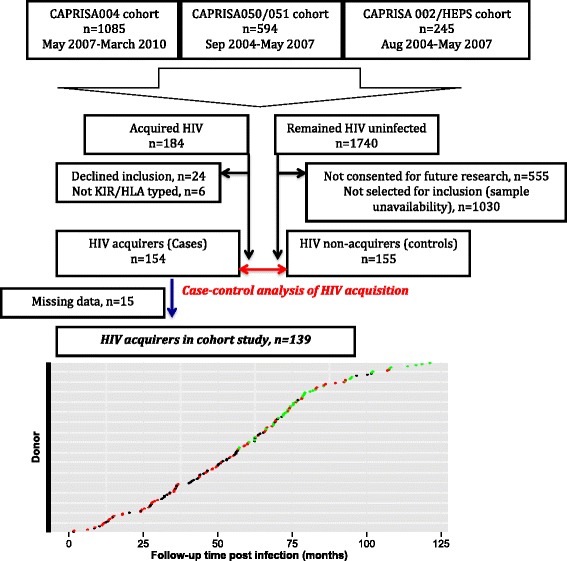
Cohort accrual diagram and study design. The figure shows the three parent studies [27–29] in which HIV negative donors were enrolled, counselled on HIV risk reduction and followed-up prospectively and how the nested case–control subset was accrued. For the case control analysis of HIV acquisition, 154 women who acquired HIV were compared with 155 women who remained HIV negative through follow-up. For cohort analyses of HIV course, 154 women who acquired HIV and were followed up for up to 120 months were studied. Follow-up time and disposition as at 1 August 2014 (date of censorship), is shown in the lower panel for each donor where dot color denotes disposition (green: remains in follow-up, red: exited study as commenced cART or death, black:lost to follow-up)

Between 2004 and 2010, we prospectively enrolled and followed-up women at risk for HIV acquisition in studies [27–29] conducted at two urban and one rural site in KwaZulu-Natal, South Africa (Fig. 1) [26]. We performed two analyses of KIR/HLA genetic profiles: a nested case–control analysis of HIV acquisition and a prospective cohort analysis of involvement in HIV disease course. For the nested case–control analysis we compared 154 women who acquired HIV to 155 who did not and for the cohort analysis, studied 139 women with viral load and CD4+ T-cell count measures.

Women at risk for HIV were extensively counselled on HIV risk reduction and provided with condoms in line with the highest standard of counselling at the time [27–29].

The median age at enrolment was 21 years (IQR 20–26 years). As previously reported [30], women who acquired HIV were younger than those who remained HIV negative (median age 23, IQR 20–26 vs. median age 33, IQR 25–40). All except one woman in this study self-identified as black-African of Zulu ethnicity.

During follow-up in the AI study, clinical care was offered as per contemporary South African treatment guidelines including combination antiretroviral therapy when clinically indicated or if the CD4+ T-cell count declined to below 200 cells/μl or 350 cells/μl (updated according to evolving treatment guidelines). Participants were seen according to the following schedule: weekly to fortnightly for the first 3 months post HIV acquisition, monthly from months 3–12 and quarterly thereafter. At each visit women received comprehensive prevention counselling, clinical examination, viral load and CD4+ T-cell count measurements and additional clinically indicated investigations performed at an accredited laboratory (ISO15189) using previously described methods [28, 29, 31]. The last possible date of censorship in those who did not reach a progression endpoint was 1 August 2014. Women were censored at time of combination antiretroviral therapy (cART) initiation, death, loss to follow-up or withdrawal from the study.

### Ethics statement

Participants gave their written and informed consent to participation in each study according to protocols approved by the Biomedical Research Ethics Committee of the University of KwaZulu-Natal (Ref 050/051, E013/04, E111/06), and relevant collaborating centres (University of Cape Town 025/2004, University of Witwatersrand M040202). The protocol under which analyses in this study are conducted was independently approved by the Biomedical Research Ethics Committee of the University of KwaZulu-Natal (BE073/10).

### KIR and HLA typing

Killer-cell immunoglobulin-like receptor genotyping was conducted in samples from 154 HIV-positive women and 155 HIV-negative women by sequence-specific oligonucleotide primer PCR or qPCR according to previously described methods [5, 32]. The definitions used to assign KIR haplotypes based on gene content were based on previous reports [7] and are described in detail in Additional file 1. Individuals having only and all genes of following group were denoted as having AA: KIR3DL3, KIR2DL3, KIR2DL1, KIR2DP1, KIR3DP1, KIR2DL4, KIR3DL1, KIR2DS4, KIR3DL2. Individuals lacking any of the following were denoted as BB: KIR2DL1, KIR2DL3, KIR3DL1, KIR2DS4. Individuals having all A haplotype genes and any 1 of the following genes were presumed to be AB: KIR2DL2, KIR2DL5, KIR2DS1, KIRDS2, KIR2DS3, KIR2DS5, KIR3DS1. Four-digit HLA typing was performed as previously described. Further information on classification of HLA alleles into Bw4 groups and C1/C2 groups is given in Additional file 1.

### Outcome definition

Methods of HIV diagnosis and estimation of the date of acquisition have been previously reported [27, 29]. Plasma HIV viral load was assessed by Roche Ampliprep/Amplicor or Roche Taqman (Roche Diagnostics, New Jersey, USA). CD4+ T-cell count enumeration in whole blood was performed by the TruCOUNT method on a FACSCalibur instrument (BD Biosciences, San Jose, USA). The median number of viral load and CD4+ T-cell count measures contributed per participant was 29 (IQR 22–34).

### Statistical methods

Statistical analyses were performed according to an *a priori* statistical analysis plan (Additional file 1) with minor amendments made to update KIR haplotype definitions. For comparisons between women who acquired HIV and those who did not, logistic regression models were employed.

To accommodate potential inter- and intra-patient sources of variation we used linear mixed-effects models to study potential correlates of HIV viral load and CD4+ T-cell count decline because of their flexibility in allowing for possible heterogeneity in population, and potential imbalance in the longitudinal data. Graphical plotting of individual measures was performed using R software, and locally weighted scatterplot smoothing (LOWESS) was employed to derive aggregate curves. Analyses were performed in R using the following packages ‘lme4’, ‘survival’, ‘ggplot2’, ‘rms’ and ‘LDheatmap’. P-values presented are nominal (unadjusted) p-values unless otherwise stated.

## Results

### Cohort accrual

Between May 2004 and March 2010, 1924 HIV negative women at risk for HIV acquisition were enrolled in one of three cohort studies in KwaZulu-Natal, South Africa and followed up to identify incident infection (Fig. 1). During a median of 16 months of active follow-up (IQR 7.8–23.7 months) with monthly or quarterly screening for HIV, 184 women acquired HIV (HIV incidence rate: 7.63 per 100 PY; 95 % CI 6.59–8.78). Of these, 160 women (of whom 154 were KIR-genotyped) consented to inclusion in continued follow-up and were enrolled at a median of 42 (IQR 28–62) days post infection into the CAPRISA AI study. As at August 2014, the median follow-up was 54 (IQR 31–69) months and at this point 8 were lost to follow up, 8 had died, 1 withdrew from the study, 101 commenced cART at a median of 50 (IQR 32–65) months after infection and 42 remain in follow-up. For cohort analyses we excluded 15 individuals due to missing data, leaving 139 individuals with follow up data. The cumulative follow-up time was 629.3 woman-years.

### Neither KIR nor HLA profiles are associated with HIV acquisition

To test whether KIR-HLA profiles are associated with risk of HIV acquisition we compared KIR gene content, and HLA alleles among 154 women (cases) who acquired HIV to 155 women (controls) who did not acquire infection during follow-up. With this sample size we had 80 % power to detect predictors with odds ratio <0.63 or >1.6 at α = 5 % given the cohort HIV incidence of ~7 %. The frequency of KIR genes, HLA alleles or groups, and KIR-HLA ligand pairs was not associated with HIV acquisition in logistic regression models (Table 1).

**Table 1:** Results of logistic regression model of KIR/HLA and HIV acquisition

	HIV- (n = 155)			HIV+ (n = 154)			p^a^	OR^a^	95 % CI^a^
	-	+	+ %	-	+	+ %			
KIR loci									
KIR2DL1	4	151	97.4 %	0	154	100.0 %	0.98	-	-
KIR2DL2	45	110	71.0 %	46	107	69.9 %	0.89	1.04	0.63–1.71
KIR2DL2/KIR2DL2		39	25.5 %		35	22.9 %			
KIR2DL2/KIR2DL3		70	45.8 %		72	47.1 %	0.63	1.15	0.43–2.05
KIR2DL3/KIR2DL3		44	28.8 %		46	30.1 %	0.83	1.07	0.32–2.00
KIR2DL3	40	114	74.0 %	35	118	77.1 %	0.61	1.15	0.68–1.94
KIR2DL4	3	152	98.1 %	0	154	100.0 %	0.98	-	-
KIR2DL5	44	111	71.6 %	56	98	63.6 %	0.18	0.72	0.44–1.16
KIR3DL1	0	155	100.0 %	0	154	100.0 %	-	-	-
KIR3DL1/KIR3DL1		145	94.8 %		141	92.2 %			
KIR3DL1/KIR3DS1		8	5.2 %		12	7.8 %	0.27	1.69	0.62-4.04
KIR3DS1/KIR3DS1		0	0.0 %		0	0.0 %	-	-	-
KIR3DS1	145	8	5.2 %	141	12	7.8 %	0.27	1.69	0.67–4.45
KIR3DL2	0	155	100.0 %	0	154	100.0 %	-	-	-
KIR3DL3	2	153	98.7 %	0	154	100.0 %	0.98	-	-
KIR2DS1	135	18	11.8 %	130	24	15.6 %	0.23	1.51	0.78–2.96
KIR2DS2	63	92	59.4 %	60	94	61.0 %	0.56	1.15	0.72–1.83
KIR2DS3	104	51	32.9 %	111	43	27.9 %	0.43	0.82	0.50–1.34
KIR2DS3/KIR2DS3		29	25.9 %		24	24.2 %			
KIR2DS3/KIR2DS5		22	19.6 %		19	19.2 %	0.77	1.13	0.49–2.60
KIR2DS5/KIR2DS5		61	54.5 %		56	56.6 %	0.77	1.11	0.57–2.14
KIR2DS5	72	83	53.5 %	79	75	48.7 %	0.47	0.85	0.54–1.33
KIR2DS4^b^	2	153	98.7 %	1	153	99.4 %	0.63	1.80	0.17–39.03
197/.		23	20.2 %		11	19.0 %			
197/219		31	27.2 %		25	43.1 %	0.25	1.57	0.70–4.21
./219		60	52.6 %		22	37.9 %	0.55	1.56	0.32–1.87
KIR Haplotype									
Bx	33	121	78.6 %	40	113	73.9 %	0.45	0.81	0.48–1.39
AA		33	21.4 %		40	26.1 %			
AB		77	50.0 %		78	51.0 %	0.65	0.88	0.50–1.55
BB		44	28.6 %		35	22.9 %	0.27	0.70	0.36–1.33
HLA Groups									
Protective B alleles	122	33	21.3 %	128	26	16.9 %	0.33	0.75	0.42–1.33
Harmful B alleles	108	47	30.3 %	104	50	32.5 %	0.80	1.07	0.66–1.73
Bw4	109	46	29.7 %	103	51	33.1 %	0.50	1.18	0.73–1.92
Bw6	75	80	51.6 %	77	77	50.0 %	0.70	0.92	0.58–1.44
C1/C1		21	13.5 %		18	11.7 %			
C1/C2		68	43.9 %		73	47.4 %	0.76	1.35	0.65–2.81
C2/C2		66	42.6 %		63	40.9 %	0.70	1.18	0.56–2.52
KIR/HLA Ligand Pair									
KIR2DL1_C2	33	122	78.7 %	25	129	83.8 %	0.22	1.44	0.81–2.59
KIR2DS1_C2	140	15	9.7 %	132	22	14.3 %	0.15	1.68	0.84–3.46
KIR2DL2_C1	90	65	41.9 %	92	62	40.3 %	0.77	0.93	0.59–1.47
KIR2DL3_C1	94	61	39.4 %	85	69	44.8 %	0.43	1.20	0.76–1.90
KIR3DL1_Bw4	109	46	29.7 %	103	51	33.1 %	0.50	1.18	0.73–1.92
KIR3DS1_Bw4	150	5	3.2 %	148	6	3.9 %	0.68	1.29	0.38–4.59
KIR2DS4_Cw04	128	27	17.4 %	128	26	16.9 %	0.86	1.04	0.57–1.91
KIR3DL2_A3A11	134	21	13.5 %	133	21	13.6 %	0.98	1.01	0.52–1.95

Previous Presentations: A part of these data was presented at the International Immunogenetics and HLA Workshop 2012. Liverpool, UK, 28 May-3 June 2012

^a^(adjusted for tenofovir gel use)

^b^For a subset (172/309, 56 %) KIR2DS4 alleles were genotyped where 197 denotes the common deletion variant of KIR2DS4 that has a 22 bp deletion in exon 5

### Presence of the KIR haplotype BB is associated with lower viral loads during HIV infection and higher CD4+ T-cell counts

To test whether KIR or HLA are associated with differences in the course of disease we used a linear mixed model approach to model post-infection viral loads accounting for differences in times of viral load or CD4+ T-cell count measurement and inter-participant variation amongst 139 women.

**Fig. 2:**
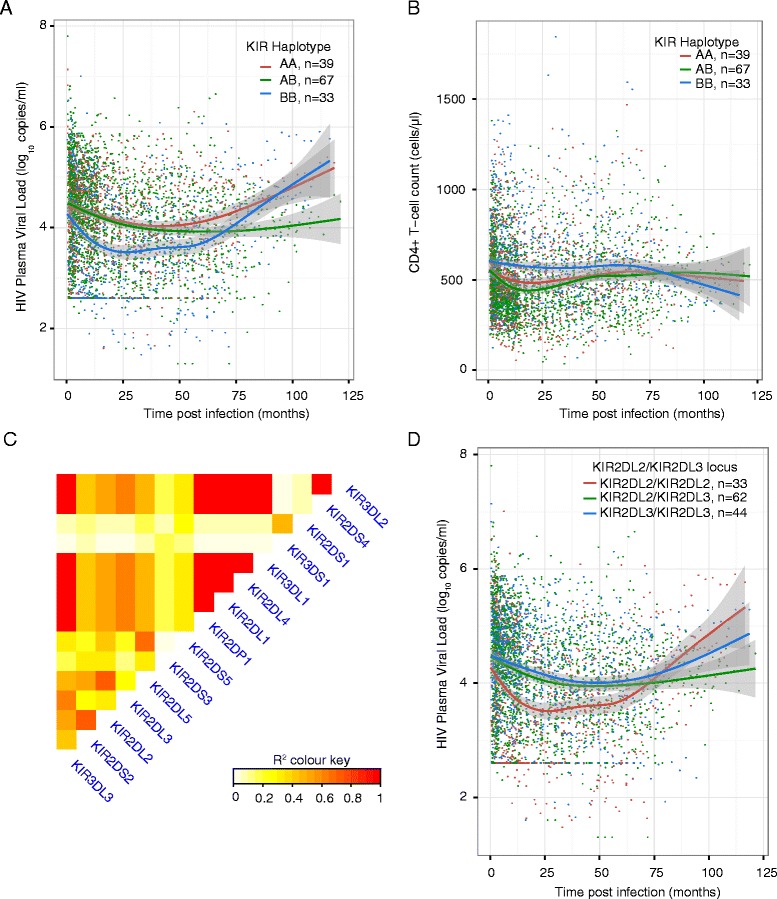
KIR haplotype BB is associated with **a** lower VL and **b** higher CD4+ T-cell counts in primary HIV infection. **c** The linkage-disequilibrium between KIR genes. **d** KIR2DL2 is associated with lower viral loads whilst KIR2DL3 (homozygous or heterozygous) is associated with relatively higher viral loads. Each plot shows the individual viral load or CD4 count measure as well as a LOWESS -smoothed curve fitted to the data as well as the 95 % confidence interval for the curve (grey shading adjacent to coloured lines)

The presence of KIR haplotype BB was significantly associated with lower viral loads (overall effect: −0.44log_10_ copies/ml, p = 0.03, Table 2 and Fig. 2a). This observation was consistent regardless of the cohort from which these women were enrolled (Additional file 2: Figure S1). Concordantly, CD4+ T-cell counts were higher in individuals with BB compared with AA/AB haplotypes (overall effect: +80 cells/μl, p = 0.04, Fig. 2b). Data beyond around 60 months of follow-up are sparse leading to overlapping confidence intervals thereafter. Cross-sectional analysis of viral loads supports this observation (Additional file 3: Figure S2). Amongst 24 women for whom CD4 + T-cell counts prior to HIV acquisition were available, the counts did not differ according to haplotype (n_AA_ = 6, n_AB_ = 14 and n_BB_ = 4; median_AA_ = 914, median_AB_ = 981 and median_BB_ = 824 cells/μl, Kruskal-Wallis rank sum test *χ* ^2^ = 1.8, p = 0.4) and the time of enrolment following HIV acquisition did not significantly differ between groups.

**Table 2:** linear mixed-effects model results for association between KIR/HLA and HIV viraemia in 139 women with incident HIV infection followed up for up to 10 years post infection

	VL Intercept (log_10_/ml)	Effect (log_10_/ml)	SE	nominal p-value
*Fixed effects*				
Haplotype				
AA	4.52	Ref	0.15	0.03
AB		−0.08	0.15	
BB		−0.44	0.18	
KIR loci				
KIR2DL2/KIR2DL2	4.08	Ref	0.15	0.02
KIR2DL2/KIR2DL3		0.33	0.16	
KIR2DL3/KIR2DL3		0.46	0.17	
KIR2DL5	4.43	−0.13	0.13	0.32
KIR3DL1/KIR3DL1	4.33	Ref	0.10	0.84
KIR3DL1/KIR3DS1		−0.05	0.24	
KIR3DS1/KIR3DS1	-	-	-	
KIR2DS1	4.36	−0.13	0.19	0.47
KIR2DS2	4.53	−0.28	0.13	0.03
KIR2DS3/KIR2DS3	4.03	Ref	0.19	0.14
KIR2DS3/KIR2DS5		0.01	0.25	
KIR2DS5/KIR2DS5		0.33	0.20	
KIR2DS4	4.43	−0.08	0.77	0.91
HLA groups				
Protective HLA-B alleles	4.38	−0.27	0.17	0.10
Harmful HLA-B alleles	4.26	0.25	0.13	0.06
Bw4	4.33	0.07	0.14	0.63
Bw6	4.39	−0.08	0.13	0.52
Number of C1 epitopes	4.40	−0.08	0.09	0.40
Number of C2 epitopes	4.25	0.07	0.09	0.43
*Random Effects*		Variance	SD	
Weeks postinfection	(Intercept)	0.02	0.12	
X002PID	(Intercept)	0.61	0.78	
Residual		0.31	0.55

Classical HLA class I alleles grouped by presence/absence of Bw4/Bw6 epitopes or C1/C2 groups were not, on their own, associated with course of viraemia but previously reported protective HLA alleles (B*13:02, B*27, B*57, B*58:01 or B*81:01) tended to be associated with lower viral loads (effect: −0.27 log_10_ copies/ml p = 0.10), and harmful alleles (B*18:01, B*35 or B*58:02) with higher viral loads (effect: +0.25 log_10_ copies/ml p = 0.06, Additional file 4: Figure S3). Inclusion of HLA alleles in the model did not attenuate association between KIR and course of HIV viraemia.

### Allelic state of KIR2DL2/KIR2DL3 may explain KIR genotype effects on HIV viral loads

Next, we examined whether the presence of specific *KIR* on the B haplotype may explain the observation above. In contrast to previous reports, the absolute number of activating or inhibitory KIR-ligand pairs did not associate with HIV viraemia (data not shown). However, the presence of *KIR2DL2* was associated with reduced HIV viral loads through the first 3–4 years of HIV and conversely, the presence of one or two copies of *KIR2DL3* was significantly associated with elevated HIV viral loads (+0.33 and +0.46 log_10_ copies/ml respectively relative to *KIR2DL2/KIR2DL2*, p = 0.02).

The extensive linkage disequilibrium between *KIR* genes (Fig. 2c), can be leveraged to further understand this observation. Contrasting direction of effect between *KIR2DL2* and *KIR2DL3* and concordant protective effect of *KIR2DS2* supports their involvement in HIV viral control because *KIR2DL2* and *KIR2DL3* segregate as alleles of the same locus and *KIR2DL2* and KIR2DS2 are in moderate linkage disequilibrium. Intriguingly, *KIR2DL2/KIR2DL3* heterozygous individuals have viral loads that are similar to *KIR2DL3/KIR2DL3* homozygous individuals suggesting that even one copy of *KIR2DL3* is associated with elevated viraemia (Fig. 2d). A similar observation is noted when examining CD4+ T-cell counts (Fig. 2b). These data are consistent with the observation that BB haplotypes of KIR are associated with reduced viraemia as *KIR2DL2* is a constituent of the B haplotype.

To assess whether KIR-ligand interactions may be involved we examined HIV viral loads according to gene content at the *KIR2DL2/KIR2DL3* locus and their ligands: HLA alleles of the C1/C2 groups (Additional file 5: Table S1). Linear mixed models testing for an interaction between *KIR2DL2/KIR2DL3* locus gene content and the number of HLA C1/C2 ligands did not provide evidence of statistical interaction. Graphical examination of viral load measures and CD4+ T-cell counts support this interpretation (Additional file 6: Figure S4). Although larger studies may reveal nuanced effects, given the observations here the association between *KIR2DL2/KIR2DL3* and HIV viraemia does not appear to be discernably modified by HLA C1/C2 ligands.

## Discussion

KIR and HLA haplotypes have been associated with HIV disease outome, but little is known about their role in HIV acquisition. Using a large cohort of prospectively enrolled women at risk for HIV, we did not observe evidence of association between KIR profiles and HIV-1 acquisition. This finding is consistent with recent studies that suggest that genetic variation does not explain a substantial proportion in liability to acquire HIV-1 [33, 34]. In contrast, we found that KIR genotype BB, which encodes KIR2DL2, was associated with lower HIV viraemia and higher CD4+ T-cell counts sustained over more than 5 years in a cohort of more than 130 prospectively followed South African women from a homogenous ethnic background.

Lack of association between KIR haplotype and HIV-1 acquisition observed here are in contrast to previous smaller studies linking higher activating:inhibitory KIR receptor repertoires, and the presence of *KIR3DS1* in particular, and genotype AB, with reduced HIV acquisition [10]. The *KIR3DS1* gene is infrequent in the population under study, which is typical for populations of African descent [35]. Our study, despite being larger than previous studies, was powered to identify covariates with unadjusted odds ratio <0.63 or >1.6, hence we may commit type 2 error if true effect sizes exist and are smaller. Although not feasible here, a more robust strategy would have been a prospective cohort analysis.

The association between KIR and HIV disease course is in agreement with immunogenetic [5] and functional studies [36] as well as recent evidence that NK cell function is a feature of HIV-1 control in patients with poor CD8+ T-cell responses [37]. Similarly, in HCV and HTLV-1 infection KIR2DL2 modifies HLA mediated effects on disease and is thereby implicated in protective responses [20]. However, our data are in contrast with observations reported by Khakoo et al. on Hepatitis C infection, where KIR2DL3 homozygous individuals had superior resolution [38]. These different outcomes may be explained by differences in pathogenesis between HCV and HIV-1. Our findings are also in contrast to a previous report of lower CD4+ T-cell counts in HIV infected KIR haplotype B carriers [39]. However, this apparent discrepancy may be due to cross-sectional sampling in Jennes et al. [39] leading to a frailty bias in selection of participants and consequent reversal of direction of effect as was recently observed in a separate study [33]. Gaudieri et al. also reported that carriage of either of the haplotype B genes KIR2DS2 or KIR2DL2 was associated with more rapid CD4+ T-cell decline, but they did not specifically assess the combined haplotype [40]. Nevertheless, these findings suggest the potential for population heterogeneity in effect and highlight the challenges in confidently delineating which KIR gene contributes to the haplotype effect.

Several underlying mechanisms may be involved in KIR2DL2 mediated enhancement of HIV control, or reciprocally of KIR2DL3 impairment. Firstly, the presence of KIR2DL2 associated footprints in virus sequenced from KIR2DL2+ donors supports a model in which KIR2DL2 may bind HIV-derived viral peptides presented by HLA-C [17]. In vitro studies using viral peptide variants suggest that selected viral peptides enhance KIR2DL2 binding, resulting in NK cell inhibition and diminished degranulation, hence affording the virus a selection advantage [41]. This may explain why the beneficial effect of KIR2DL2 may be lost later in infection. Whilst differing by only a few amino acids, KIR2DL2 has been reported to have higher affinity for HLA-CI than KIR2DL3, and KIR2DL2/2DL3 differ in their sensitivity to peptide bound in the HLA C groove offering a further potential explanation for how subtle differences may explain the divergent effects of KIR2DL2/L3 [42]. Although Korner et al. [15], show that KIR2DL2+ NK cells are functionally more potent in the presence of HLA-C1/C1, because KIR2DL2 mediates inhibitory signals following binding to HLA-C molecules of both HLA-C1 or HLA-C2, the absence of an interaction between KIR2DL2 and HLA-C1/C2 group in our study is compatible with this model [43]. The linkage disequilibrium that we found between KIR2DL2 and KIR2DS2 was consistent with previous studies [44] and implies that a role for KIR2DS2 cannot be excluded. An alternative mechanism is suggested by Schonberg et al. [43] who report that due to differences in timing of KIR2DL2 and KIR2DL1 expression, the presence of KIR2DL2 may affect ligand-instructed NK cell education during development. Finally, KIR2DL2 may act indirectly to alter T-cell recognition of virally infected cells as described in HTLV-1 and HCV infection [20].

Blockade of inhibitory KIR interaction with HLA-C has been pursued as therapeutic strategy in malignancy and viral infection [45]. An antibody that blocks interaction of KIR2DL1/L2/L3/S1/S2 (1-7 F9) with HLA-C, was shown to enhance degranulation of NK cells from HIV infected donors cocultured with target cells in vitro [46]. The enhancement in NK cell degranulation observed with 1-7 F9 was higher amongst NK cells from donors with KIR haplotype B in congruence with our observations. However, precise delineation of the role of KIR2DL2, KIR2DL3 and KIR2DS2 is difficult. This approach has been further developed in acute myeloid leukaemia (AML), multiple myeloma (MM) and lymphoma models [47–49]. A humanised version (lirilumab) was shown to be safe [50] in humans and is currently in clinical trials for treatment of AML (NCT01687387), MM (NCT02252263, NCT01592370), lymphoma (NCT01592370) and some solid organ tumours (NCT01750580, NCT01714739). We speculate that similar approaches may be beneficial in HIV.

In spite of having a pre-determined data analysis plan and consistent viral load and CD4+ T-cell measures across both sub-cohorts, we cannot exclude that this finding is a false positive due to multiple comparisons. Larger studies, that limit analyses to replication of this finding, are required. In addition, this study has other noteworthy limitations. Firstly, it is limited by the lack of resolution of specific KIR alleles and copy number. Secondly, in the case control analysis we were unable to account for the transmitting partners KIR status constraining our ability to confirm previous reports of KIR2DS4*001 carriage in the transmitting partner being linked to enhanced transmission risk [51]. Thirdly, the limited sample size prohibited subgroup analyses, for example, of HLA C1/C2 ligand interactions. Finally, we were unable to link these clinical observations with previously reported flow-cytometry based measures of NK cell function [23] due to the absence of KIR2DL2/L3 specific antibodies used in our previous work.

## Conclusions

Our data suggest that *KIR2DL2* on the B KIR haplotype may mediate measurable control of HIV viraemia and underlies a protective effect through the early years of chronic HIV infection. Larger studies are required to confirm this finding and establish its generalizability. These data also support exploration of using KIR-blocking approaches to manipulate NK cell function in HIV infection.

## Additional files

Additional file 1: **Statistical Analysis plan: KIR/HLA and viral control in seroconverters in CAPRISA 004 and CAPRISA050/051/HEPS.** (DOCX 23 kb)

Additional file 2: Figure S1. KIR haplotype BB is associated with lower viral loads in both subsets of the cohort as shown in upper (CAPRISA 050/051 and CAPRIS002/HEPS) and lower (CAPRISA004) panels respectively. Upper and lower panel shows individual viral load measures (dots) and LOWESS smoothed curve coloured according to donor KIR haplotype with 95 % CI shown in grey shading. (PDF 1119 kb)

Additional file 3: Figure S2. Cross-sectional viral load measures according to KIR haplotype. Boxes denote interquartile range (box margin) and median (solid line), and whiskers denote range. (PDF 1210 kb)

Additional file 4: Figure S3. Viral load measures according to HLA-types. . Each panel shows individual viral load or CD4+ T-cell count measures (dots) and LOWESS smoothed curve coloured according to HLA classification as protective, harmful or reference according to previous studies, with 95 % CI shown in grey shading. (PDF 1148 kb)

Additional file 5: **Supplementary Table 1.** Distribution of KIR2DL2/KIR2DL3 allele status according to HLA-C ligand groupings. (DOCX 38.3 kb)

Additional file 6: Figure S4. Viral load (A-C) and CD4+ T-cell count (D-F) measures in KIR2DL2/KIR2DL2 (A, D), KIR2DL2/KIR2DL3 (B, E), KIR2DL3/KIR2DL3 (C, F) according to HLA-C1/HLA-C2 ligand grouping. Each panel shows individual viral load or CD4+ T-cell count measures (dots) and LOWESS smoothed curve coloured according to donor HLA C1/C2 allele groupings. (PDF 1822 kb)
