# Supplementary material for: Killer-cell Immunoglobulin-like Receptor (KIR) gene profiles modify HIV disease course, not HIV acquisition in South African women
Source: BMC Infect Dis. 2016 Jan 25;16:27. doi: 10.1186/s12879-016-1361-1 (PMC4727384; doi:10.1186/s12879-016-1361-1)
Supplement: Supplementary file 1 — Statistical Analysis plan: KIR/HLA and viral control in seroconverters in CAPRISA 004 and CAPRISA050/051/HEPS. (DOCX 23 kb) [file 12879_2016_1361_MOESM1_ESM.docx]

**Statistical Analysis plan: KIR/HLA and viral control in seroconverters in CAPRISA 004 and CAPRISA050/051/HEPS**

**14 May 2010**

*update September 2014: survival analyses section removed en masse.*

**Aim:** To determine whether:

1. Specific KIR haplotypes predict/associate with outcomes of HIV infection
2. Specific KIR associate with outcomes of HIV infection
3. Specific HLA groups (Bw4/Bw6) associate with outcomes of HIV infection
4. Specific HLA associate with outcomes of HIV infection
5. HLA haplotypes associate with outcomes of HIV infection
6. KIR-HLA compound genotypes associate with outcomes of HIV infection

**Statistical Approach:**

**A logistic regression for HIV acquisition**

**B Mixed linear regression for viremia outcome** OUTCOME=continuous:

1. linear mixed model, repeated measures)
2. Censor individuals when treatment commences/LTFU/death

Alternative considerations (not performed but discussed)

- Mean log viral load over between 9-15 months OR
- Mean during the period for which the first and last viral load is within 0.25log of the mean log viral loads in between.

Run logistic and mixed linear regression for

- KIR haplotype (AA, AB, BB, and for the Bx breakdown by C4/T4 groups)
- Specific KIR (test each with frequency<100%)or combinations thereof (Number of activating KIR, ratio of activating to inhibitory KIR’s)
- HLA by Bw4/Bw6 groups
- Specific HLA-Alleles (Use macro approach to select)
- HLA haplotypes (definitions from Debbie)
- KIR-HLA ligand match vs mismatch (enumerate number of matches and numbers).

**Methods**

1. **Define KIR haplotypes:**

Classify by the following: any unclassifiable-mark as such.

1. Patients having only and all genes of following group = **AA**

{KIR3DL3, KIR2DL3, KIR2DL1, KIR2DP1, KIR3DP1, KIR2DL4, KIR3DL1, KIR2DS4, KIR3DL2}

1. Patients lacking any of the following=**BB**

KIR2DL1, KIR2DL3, KIR3DL1, KIR2DS4

1. Patients = **AB** if:

Have ALL OF {KIR3DL3, KIR2DL3, KIR2DL1, KIR2DP1, KIR3DP1, KIR2DL4, KIR3DL1, KIR2DS4, KIR3DL2}

*AND* >=1 of

{KIR2DL2, KIR2DL5,KIR2DS1, KIRDS2, KIR2DS3, KIR2DS5, KIR3DS1}

IF AB OR BB then code as Bx (All AB or BB are Bx.)

If Bx then Define **Bx** **Gene Arrangemen**t—

If a donor has any of the four genes indicated for C4 or for T4 then classify as such.

C4 (Centromeric 4)= *KIR2DS2-2DL2-2DS3-2DL5*

T4 (Telomeric 4)= *KIR3DS1-2DL5-2DS1-2DS5*

Code as :

- C4/T4
- Cx/T4
- C4/Tx
- Cx/Tx

Where x= not present (code into 4 groups based on presence or absence of C4/T4)

1. **Define HLA groups:**

HLA B (and A):

(HLA typing is by two or four digits. Below are the two/four digit types that classify into Bw4 or Bw6. Hence if B58 then Bw4; full four digit can be B5801, B5802, B5811 etc. )

**Bw4:**

B05, B5102, B5103, B13, B17, B27, B37, B38, B44, B47, B49, B51, B52, B53, B57, B58, B59, B63, B77

and A09, A23, A24, A2403, A25, A32

**Bw6:**

B07, B703, B08, B14, B18, B22, B2708, B35, B39, B3901, B3902, B40, B4005, B41, B42, B45, B46, B48, B50, B54, B55, B56, B60, B61, B62, B64, B65, B67, B70, B71, B72, B73, B75, B76, B78, B81, B82

Code each donor for each of the two HLA B alleles (as separate variables) and then:

- as **Bw4/Bw4** if both HLA-B are Bw4,
- As **Bw6/Bw6** if both HLA-B are Bw6
- As **Bw4/Bw6** if patient has one Bw4 and one Bw6 HLA-B

HLA C:

(HLA typing is by two or four digits. Below are the two/four digit types that classify into C1 or C2. Hence if C*07 then C2; full four digit can be C0702, C0701 etc.)

**C1** supergoup: C*01, C*03, C*07, C*08

**C2** supergroup: C*02, C*04, C*05, C*06

Code each donor for each of the two HLA-C alleles (as separate variables) and then

- as **C1/C1** if both HLA-C are C1,
- As **C2/C2** if both HLA-C are C2
- As **C1/C2** if patient has one C1 and one C2 HLA-C

1. **KIR-HLA ligand match**

For each of the following KIR, check whether a matching ligand exists or not. Create a variable for each KIR group indicated and code whether matching or not.

KIR2DL1 HLA-C C2

KIR2DS1 HLA-C C2

KIR2DL2 HLA-C C1/C2

KIR2DL3 HLA-C C1

KIR2DS2 HLA-C C1

KIR3DL1/S1 HLA-B Bw4

KIR3DS1 HLA-B Bw4

KIR2DS4 HLA-C*04 (C*04 only, not any other C2)

KIR3DL2 HLA A A11 or A3 (???)
