# Supplementary figures and images for: Killer-cell Immunoglobulin-like Receptor (KIR) gene profiles modify HIV disease course, not HIV acquisition in South African women
Source: BMC Infect Dis. 2016 Jan 25;16:27. doi: 10.1186/s12879-016-1361-1 (PMC4727384; doi:10.1186/s12879-016-1361-1)

A

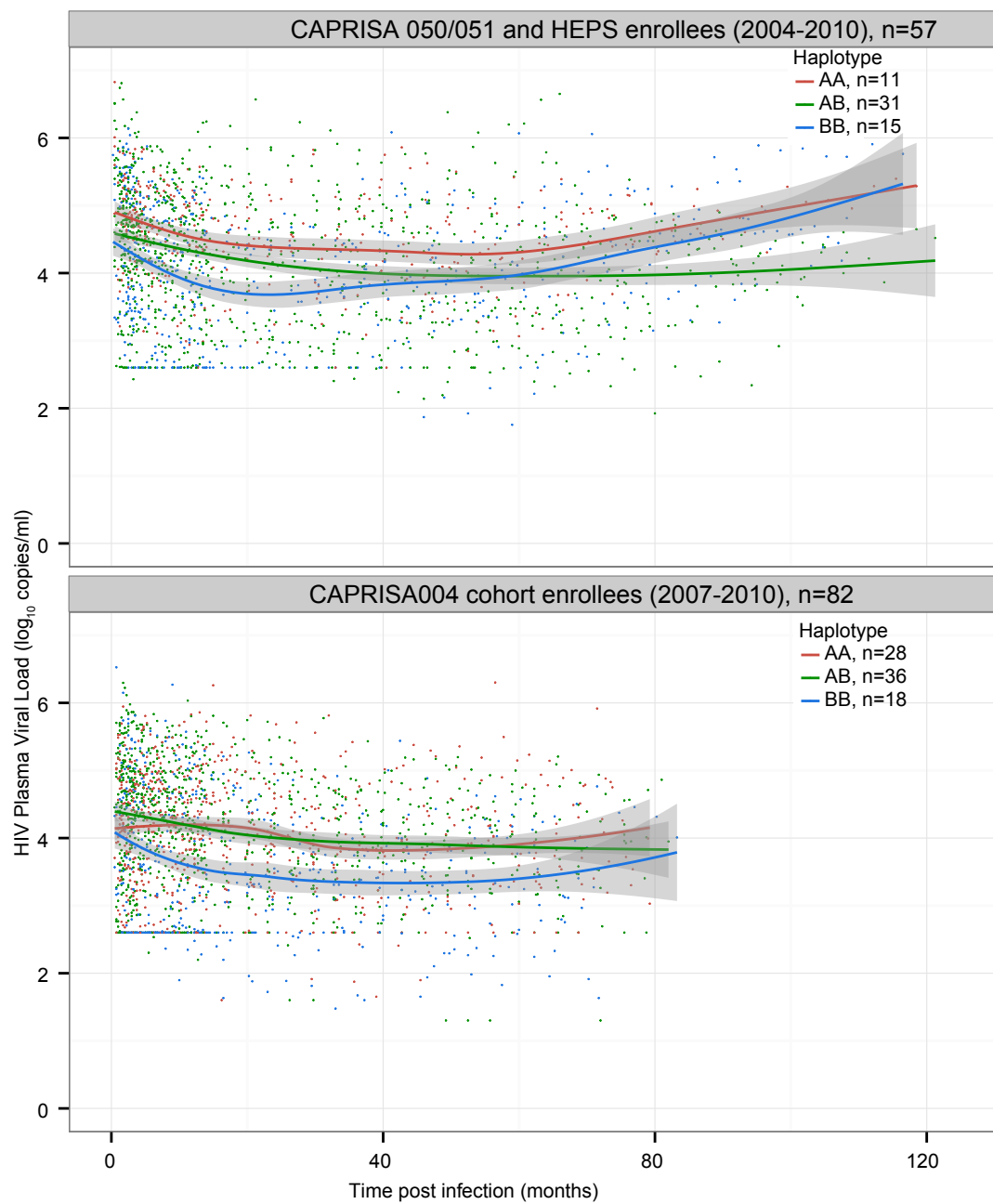

Supplement: Supplementary file 2 — KIR haplotype BB is associated with lower viral loads in both subsets of the cohort as shown in upper (CAPRISA 050/051 and CAPRIS002/HEPS) and lower (CAPRISA004) panels respectively. Upper and lower panel shows individual viral load measures (dots) and LOWESS smoothed curve coloured according to donor KIR haplotype with 95 % CI shown in grey shading. (PDF 1119 kb) [file 12879_2016_1361_MOESM2_ESM.pdf]

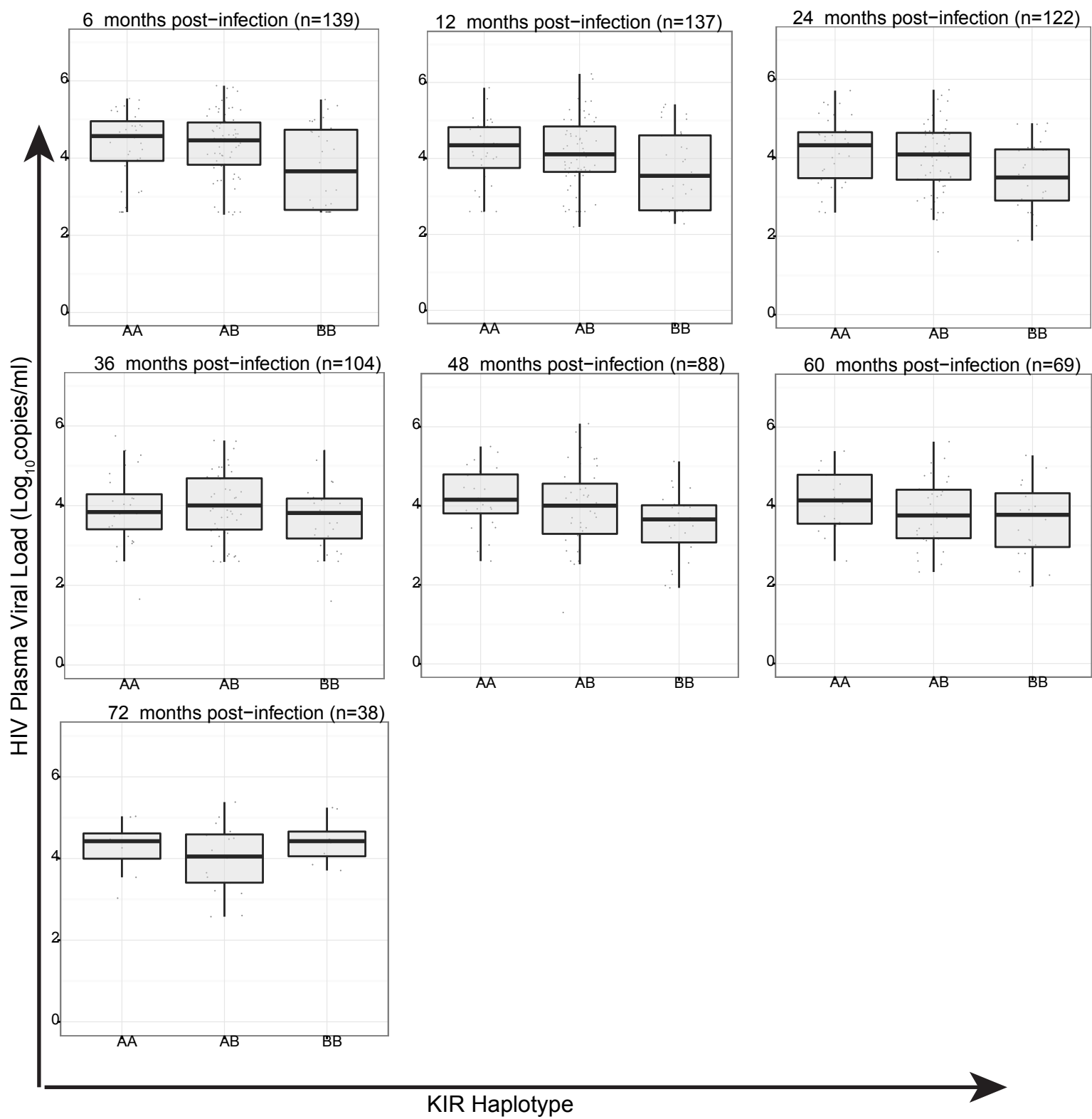

Supplement: Supplementary file 3 — Cross-sectional viral load measures according to KIR haplotype. Boxes denote interquartile range (box margin) and median (solid line), and whiskers denote range. (PDF 1210 kb) [file 12879_2016_1361_MOESM3_ESM.pdf]

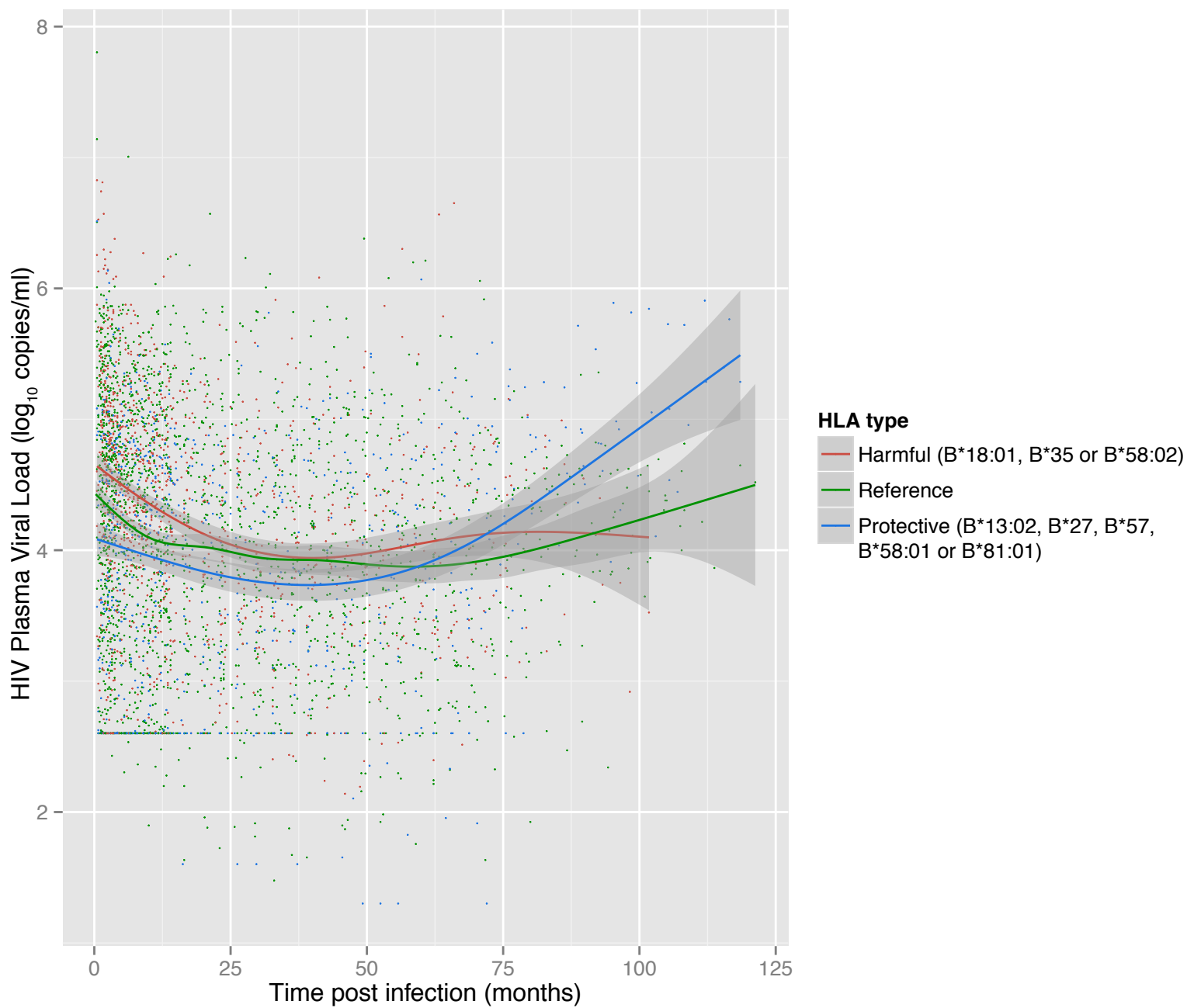

Supplement: Supplementary file 4 — Viral load measures according to HLA-types. . Each panel shows individual viral load or CD4+ T-cell count measures (dots) and LOWESS smoothed curve coloured according to HLA classification as protective, harmful or reference according to previous studies, with 95 % CI shown in grey shading. (PDF 1148 kb) [file 12879_2016_1361_MOESM4_ESM.pdf]

A

KIR2DL2/KIR2DL2

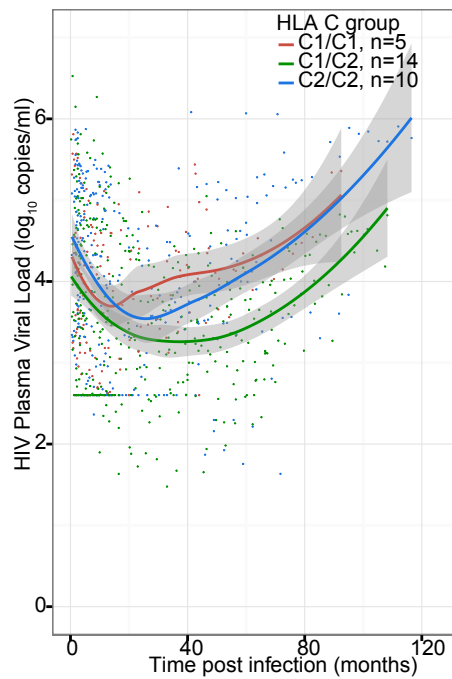

B

KIR2DL2/KIR2DL3

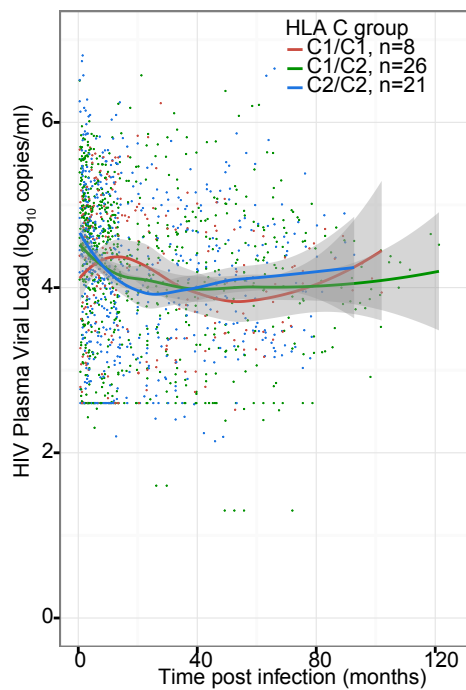

C

KIR2DL3/KIR2DL3

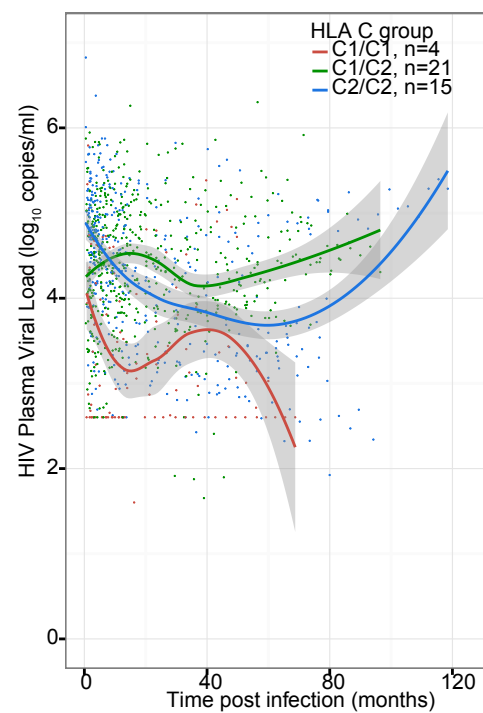

D

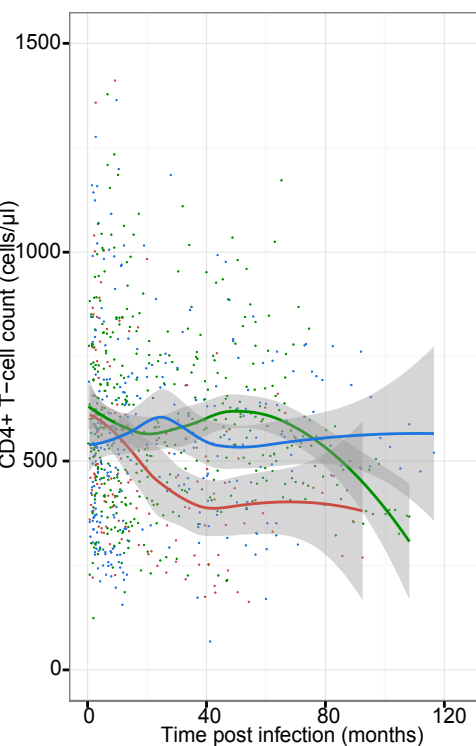

E

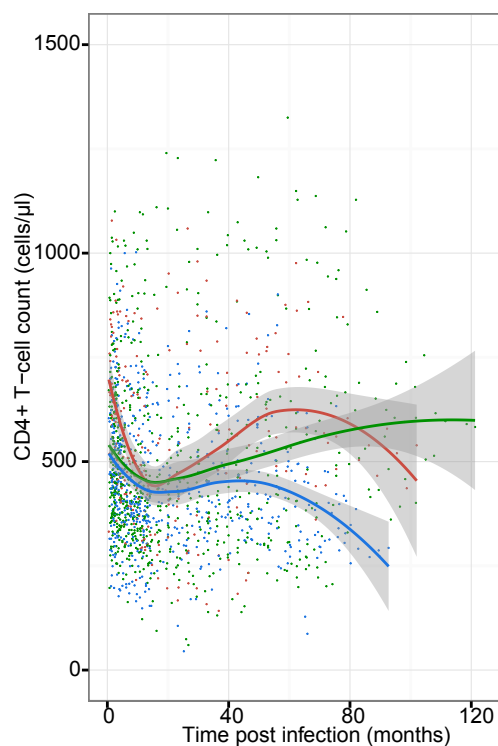

F

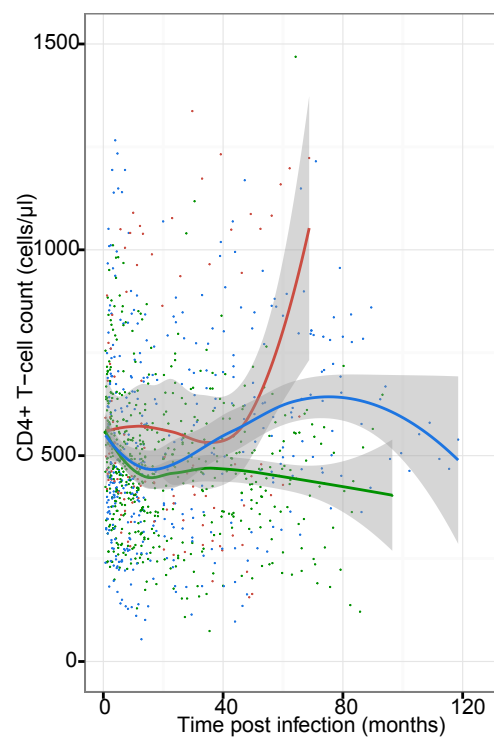

Supplement: Supplementary file 6 — Viral load (A-C) and CD4+ T-cell count (D-F) measures in KIR2DL2/KIR2DL2 (A, D), KIR2DL2/KIR2DL3 (B, E), KIR2DL3/KIR2DL3 (C, F) according to HLA-C1/HLA-C2 ligand grouping. Each panel shows individual viral load or CD4+ T-cell count measures (dots) and LOWESS smoothed curve coloured according to donor HLA C1/C2 allele groupings. (PDF 1822 kb) [file 12879_2016_1361_MOESM6_ESM.pdf]
