# Supplementary material for: Killer-cell Immunoglobulin-like Receptor (KIR) gene profiles modify HIV disease course, not HIV acquisition in South African women
Source: BMC Infect Dis. 2016 Jan 25;16:27. doi: 10.1186/s12879-016-1361-1 (PMC4727384; doi:10.1186/s12879-016-1361-1)
Supplement: Supplementary file 5 — Supplementary Table 1. Distribution of KIR2DL2/KIR2DL3 allele status according to HLA-C ligand groupings. (DOCX 38.3 kb) [file 12879_2016_1361_MOESM5_ESM.docx]

Supplementary Table 1: Distribution of KIR2DL2/KIR2DL3 allele status according to HLA-C ligand groupings

| HLA-C Group | KIR2DL2/KIR2DL3 locus status | | |
| --- | --- | --- | --- |
|  | KIR2DL2/KIR2DL2 | KIR2DL2/KIR2DL3 | KIR2DL3/KIR2DL3 |
| C1/C1 | 5 | 14 | 10 |
| C1/C2 | 14 | 26 | 21 |
| C2/C2 | 10 | 21 | 15 |
